# Seismic response analysis of double-trough aqueduct considering fluid-structure interaction effect

**DOI:** 10.1371/journal.pone.0289600

**Published:** 2023-08-04

**Authors:** Liang Huang, Haotian Li, Mwansa Andrew Lupunga, Jianguo Xu, Chunyu Zhang

**Affiliations:** School of Water Conservancy and Civil Engineering, Zhengzhou University, Zhengzhou, China; Universiti Teknologi Malaysia, MALAYSIA

## Abstract

At present, the crude fluid-structure interaction analysis model cannot accurately characterize the interaction mechanism between aqueduct and water under earthquake action. In order to solve this problem, this paper analyzes the seismic response of the double-tank aqueduct under the action of earthquake by using the shaker test and the VOF (Volume of Fluid) method considering the free liquid level from the perspective of fluid-solid bidirectional coupling, explores whether the liquid movement in the double tank is consistent and the shock absorption effect of different water levels on the aqueduct, and analyzes the amplitude of free liquid level sloshing and the change of horizontal dynamic pressure caused by water level change from the generation mechanism of TLD (Liquid tuning dampers). The results show that the liquid movement in the two tanks in the double-channel aqueduct is basically the same under the action of earthquake, and the TLD effect of the liquid gradually increases with the increase of the water level in the aqueduct, and the maximum peak shock absorption rate is 63.4% at the maximum peak and 50.4% in numerical simulation. The shaking amplitude of the liquid is positively correlated with the water level height, and the magnitude of the shaking amplitude also reflects the magnitude of the moving water pressure.

## 1. Introduction

China is a country with abundant water resources, but at the same time, it is a country with water shortages per capita. Due to this imbalance of water resources, water shortages in some areas have been perennial, seriously affecting the livelihood and economic development of local people. China has built a series of joint water resource management projects to improve the uneven distribution of water resources in the country’s regions. In many water transmission and diversion projects, the aqueduct is a very widely used cross-building. Despite the aqueduct structure span being large, the anti-side stiffness is low, and the upper trough body carries a large mass that might even exceed its own weight causing the whole structure to form a very unfavorable seismic "top-heavy" form. The interaction between the water body and the structure also affects the response of the entire structure under the action of an earthquake, so it is necessary to conduct a seismic analysis of the aqueduct structure and study the TLD effect of the water body in it.

When considering coupling to analyze the dynamic characteristics of aqueduct structures, many scholars mostly use additional mass or spring-mass models for analysis. Among them, Wang Bo et al. [1] proposed a spatial dynamic analysis model of the thin-walled structure of the aqueduct according to the thin-walled member structure of the aqueduct body and analyzed the dynamic characteristics of the aqueduct by superimposing the water body in the trough according to the concentrated mass on the corresponding trough node. Li Zhengnong et al. [2] analyzed the self-vibration characteristics of a multi-trough aqueduct by applying three-dimensional dynamic finite elements, and the influence of water mass in the trough was calculated into the corresponding nodes in the form of additional mass, and the self-vibration characteristics of the structure were analyzed by mode decomposition method. Wu Yi et al. [3] carried out a modal analysis of the aqueduct structure considering the interaction between fluid and structure, and concluded that the water body influences the self-resonant frequency of the aqueduct structure and the higher the water level in the aqueduct structure, the smaller the self-resonance frequency of the aqueduct structure through the water. When Liu Yunhe et al. [4] studied the application of the Housner spring-mass model to the seismic fluid-structure interaction analysis of aqueduct structure, they pointed out that the analysis object is a flexible aqueduct, and it would be dangerous to use the Housner model. Li Yuchun et al. [5] adopted linear potential flow theory and analogy method in the existing classical theory, mathematically simplified the obtained exact solution formula, and proposed a suggested formula with simple expression and high precision.

With the development of finite element software and the improvement of computer computing power, many scholars can more comprehensively analyze the boundary conditions and influencing factors of fluid-structure interaction models. Shao Yan [6] used ANSYS to establish the aqueduct model, used Visual Fortran to perform fluid-structure interaction, calculated the dynamic response of the aqueduct model, and then finally compared the calculation results of the three methods. Wang Bo [7] also considered the fluid-structure interaction and pile-soil interaction, and then used ABAQUS software to establish a three-dimensional simulation mechanical model of pile-soil-aqueduct-water to analyze the dynamic characteristics. Reza Kamgar [8] uses numerical solutions to conclude that the seismic design of an modified tuned liquid damper system (MTLD) can more effectively reduce the response of the structure. Alemdar Bayraktar et al. [9] studied the effect of fluid-structure interaction on the improvement of seismic performance of historical masonry aqueducts, compared the water-bearing aqueduct model with the waterless aqueduct model, and concluded that a certain water level can reduce the damage of the historical aqueduct structure under earthquake conditions. Mordanova and De Felice [10] determined collapse mechanisms of Claudio Aqueduct in Rome for actual and reinforced states under earthquake loads using discrete element method.

Li Zhengnong [11] combined with the Huohe Aqueduct in the middle line of the South-to-North Water Diversion Project, used a single-span overall structural model with a geometric ratio of 1/35 to conduct a shaker test, which included the foundation, trough pier and trough body, to study the seismic response of the structure. Jiang Yinjun [12] also combined with the Huohe aqueduct, using a single-span model with a geometric ratio of 1/30 to carry out the shaker test research, and the aqueduct is a rectangular section of three troughs, the seismic response of the structure in the unsupported working condition and the three different stiffness damping support conditions were tested respectively. Zhang Linrang [13] took the yellow box-shaped aqueduct of the South-to-North Water Diversion Project as the research object, and used the hydroelastic vibration rectangular section aqueduct model with a geometric ratio of 1/27.8 to carry out experiments on the horizontal shaking table, and studied the interaction effect and mechanism of both water body and trough under seismic action and sine wave excitation. By analyzing the dynamic response of the aqueduct structure under white noise excitation and bidirectional seismic wave excitation, Wang Haibo et al. [14] determined the important influence of the water body in a large thin-walled aqueduct on the self-resonance frequency. The structure and the supporting force at both ends of the aqueduc gave the equivalent mass quantitative relationship of the dynamic interaction between the water body and the aqueduct structure of the test subject.

**Fig 1:**
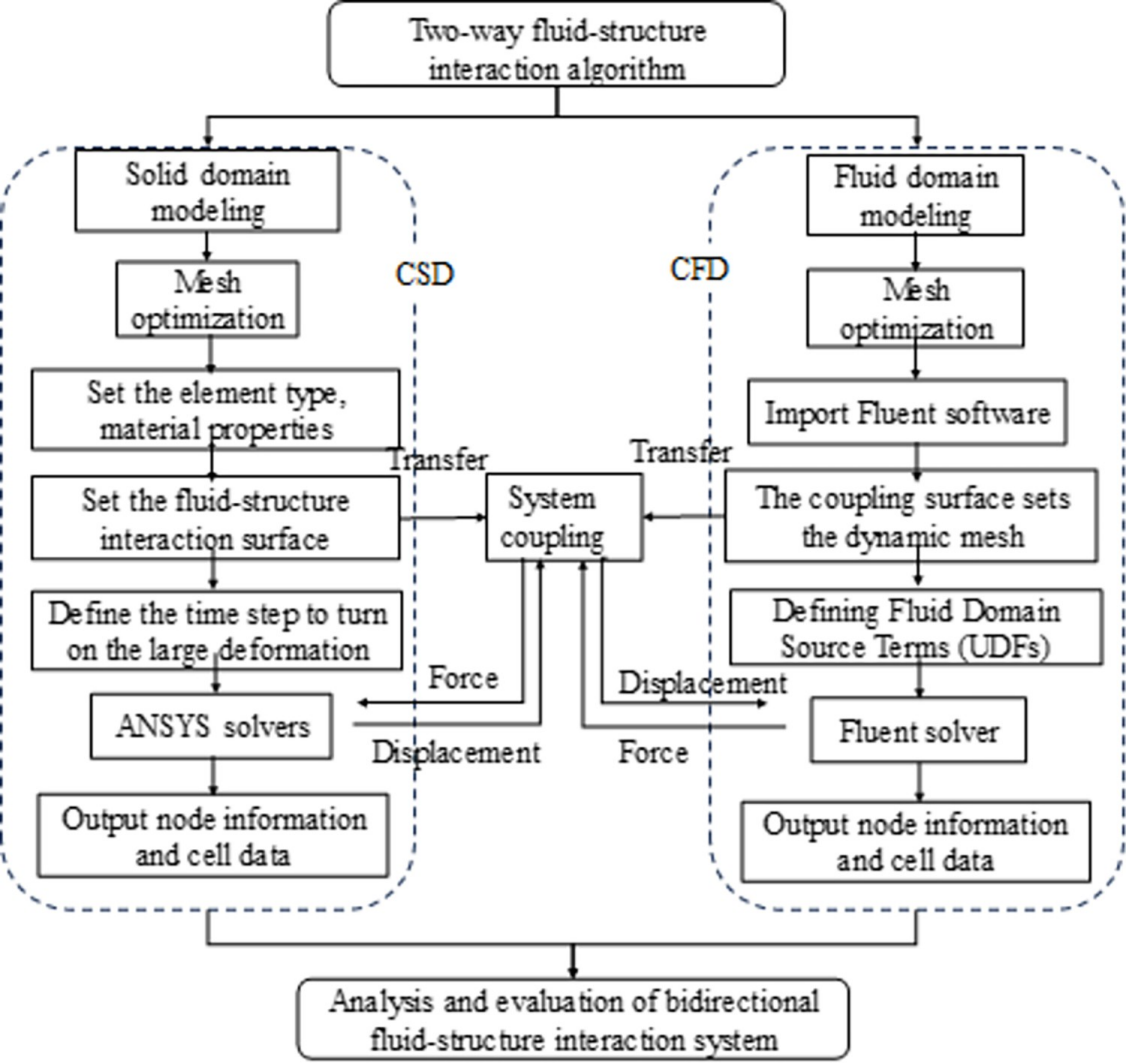
Two-way fluid-structure interaction process.

Studying the mechanism and influencing factors of fluid-structure interaction of aqueducts is of great engineering significance for accurately analyzing the seismic response of aqueduct structures and ensuring the seismic safety of aqueduct structures, but most of the existing research is only aimed at static analysis and dynamic analysis of aqueducts, and there are few studies on aqueduct fluid-structure interaction, especially bidirectional coupling, and little research on the mechanism of fluid in aqueducts. In this paper, the coupling method of zonal solution is innovatively proposed to establish the aqueduct model, and the VOF method is used to analyze the shaking effect of the free liquid level in the aqueduct fluid domain, and the data transmission of the bidirectional coupling algorithm is closer to the dynamic response of the aqueduct structure under the actual earthquake. In this paper, an aqueduct shaker test with liquid is also carried out to explore the dynamic response of the aqueduct model at different water levels. The numerical model carried out the TLD effect under fluid-structure interaction from the aspects of aqueduct displacement response, wall hydrodynamic pressure, free liquid level change, and self-resonance frequency. The flowchart of the two-way fluid-structure interaction algorithm used in this paper is shown in the Fig 1

## 2. Numerical simulation methods

Computational Fluid Dynamics (CFD) is a branch of fluid mechanics that uses discretized numerical methods to numerically simulate and analyze fluid motion and its mechanical characteristics. In general, the flow of fluids obeys the laws of conservation of physics, including the law of conservation of mass, the law of conservation of momentum, and the law of conservation of energy. Without considering heat exchange, no energy conservation equations are involved [15].

(1) Mass conservation equation

The law of conservation of mass represents the increase in mass in a fluid microelement per unit time equal to the net mass flowing into the microbody over the same time interval. Considering that the fluid is incompressible and the density is constant, the mass conservation equation is:

$$\frac{\partial u}{\partial x}+\frac{\partial v}{\partial y}+\frac{\partial w}{\partial z}=0$$ (1)

where u, v, and w are velocity in the x, y, z directions, respectively and x,y,z is the spatial position of the fluid particle.

(2) Momentum conservation equation

The law of conservation of momentum is expressed as the rate of change in time of the momentum of a fluid in a microelement body equal to the sum of the forces acting on the microelement body by the outside world. For incompressible fluids, the N-S equation is expressed as a tensor as follows:

$$\rho \frac{du}{dt}=-\nabla p+\rho g+\nabla \cdot 2\mu S+\nabla (\lambda \nabla \cdot u)$$ (2)

where p is the pressure on the fluid control body, λ is the bulk expansion viscosity coefficient of the fluid, μ is the dynamic viscosity coefficient of the fluid, ρ is the density of the fluid. The components of the velocity vector u in the three directions of x, y, z are u, v, w, g is the acceleration vector of the control body.

There are two ways [16] to simulate liquid level shaking: rigid body motion method, where equations are solved in an inertial reference frame, and meshes move in reality; Momentum source method, grid fixation, equations solved in a motion reference frame. In this study, the momentum source method is used, that is, the law of conservation of physics under non-inertial coordinate systems is considered. The expression for the N-S equation in a non-inertial coordinate system is:

$$\rho \frac{du}{dt}=-\nabla p+\rho g+\nabla \cdot 2\mu S+\nabla (\lambda \nabla \cdot u)-\rho (a_{2}+\dot{\omega }R+\omega \times \omega R+2\omega u)$$ (3)

Where: ***a***_*R*_ is the moving implicated acceleration, that is, the displacement acceleration of the non-inertial coordinate system relative to the inertial coordinate system, $\dot{\omega }\times R$, *ω*×*ω*×*R* respectively, rotational implicated acceleration and rotational implicated centripetal acceleration, that is, the acceleration generated by the rotation of the liquid particle relative to the non-inertial coordinate system, 2*ω*×*u* is Coriolis acceleration.

(3) Volume of Fluid (VOF) method

The VOF model is a type of multiphase flow model that is primarily used to track the interface position of two or more incompatible fluids [17–19]. The fluid volume method (VOF) volume function equation is:

$$\frac{\partial F}{\partial x}+\nabla \cdot (uF)=0$$ (4)

where u is the speed (m/s), F is the fluid volume fraction and t is the time (s).

### 2.1 Fluid-structure interaction methods

At the fluid-structure interaction interface, continuous conditions should be met, that is, the stresses and displacements of fluids and solids should be equal:

$$\begin{array}{c} \sigma_{f}\times n_{f}=\sigma_{s}\times n_{s}\\ v_{t}=v_{s} \end{array}$$ (5)

where *n*_*f*_ is the normal unit vector of the fluid domain at the fluid-structure interaction interface, *n*_*s*_ is the normal unit vector of the solid domain at the fluid-structure interaction interface.

**Fig 2:**
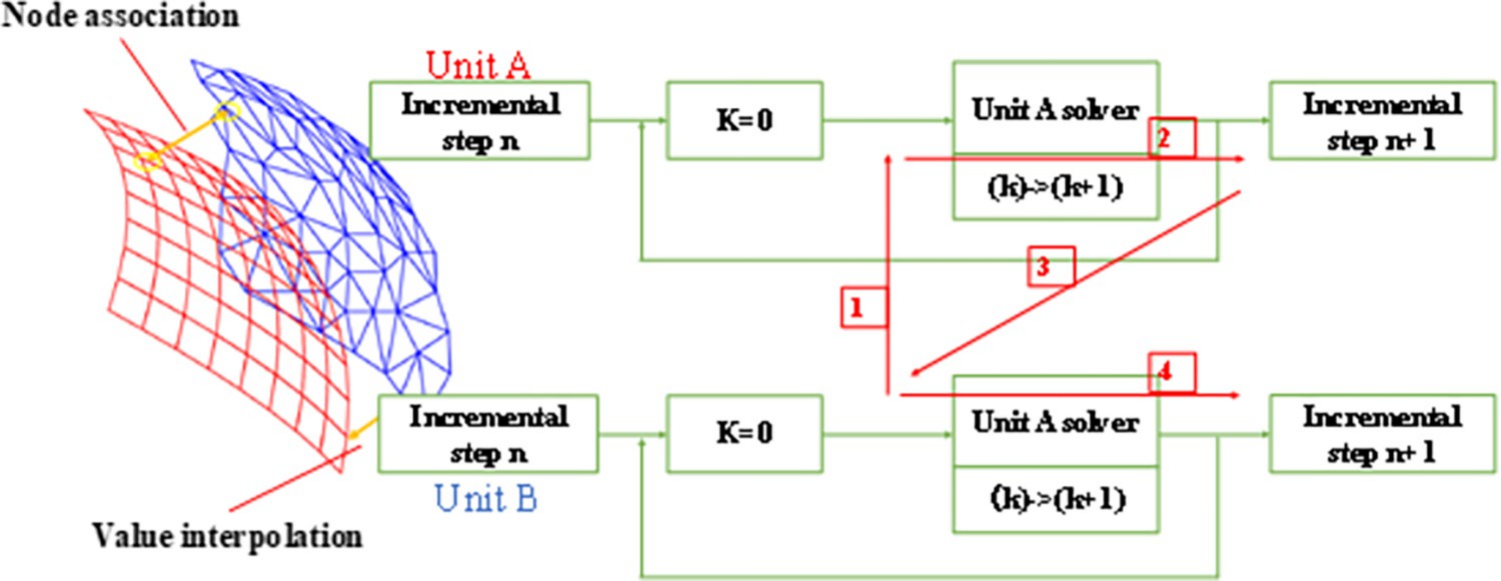
Bidirectional fluid-structure interaction process.

Unidirectional fluid-structure interaction means that the direction of data transfer at the fluid-structure interface is unidirectional, usually transferring the calculation results of the fluid to the solid. Unidirectional fluid-structure interaction is generally used when the fluid has a significant effect on the solid, while the solid has a negligible effect on the fluid. Bidirectional fluid-structure interaction [20, 21] means that the direction of data transfer is bidirectional, which is applied when fluids and solids affect each other. The bidirectional fluid-structure interaction algorithm (Fig 2) of zonal solution does not need to consider the fluid-structure interaction control equation. Through a predefined order, the fluid problem and the solid problem are calculated independently in the fluid solver and the solid solver respectively, and then the calculation results are exchanged and passed using the fluid-structure interface. If the convergence within this time step meets the requirements, we can move forward until the finally result is obtained.

### 2.2 Numerical modelling

**Fig 3:**
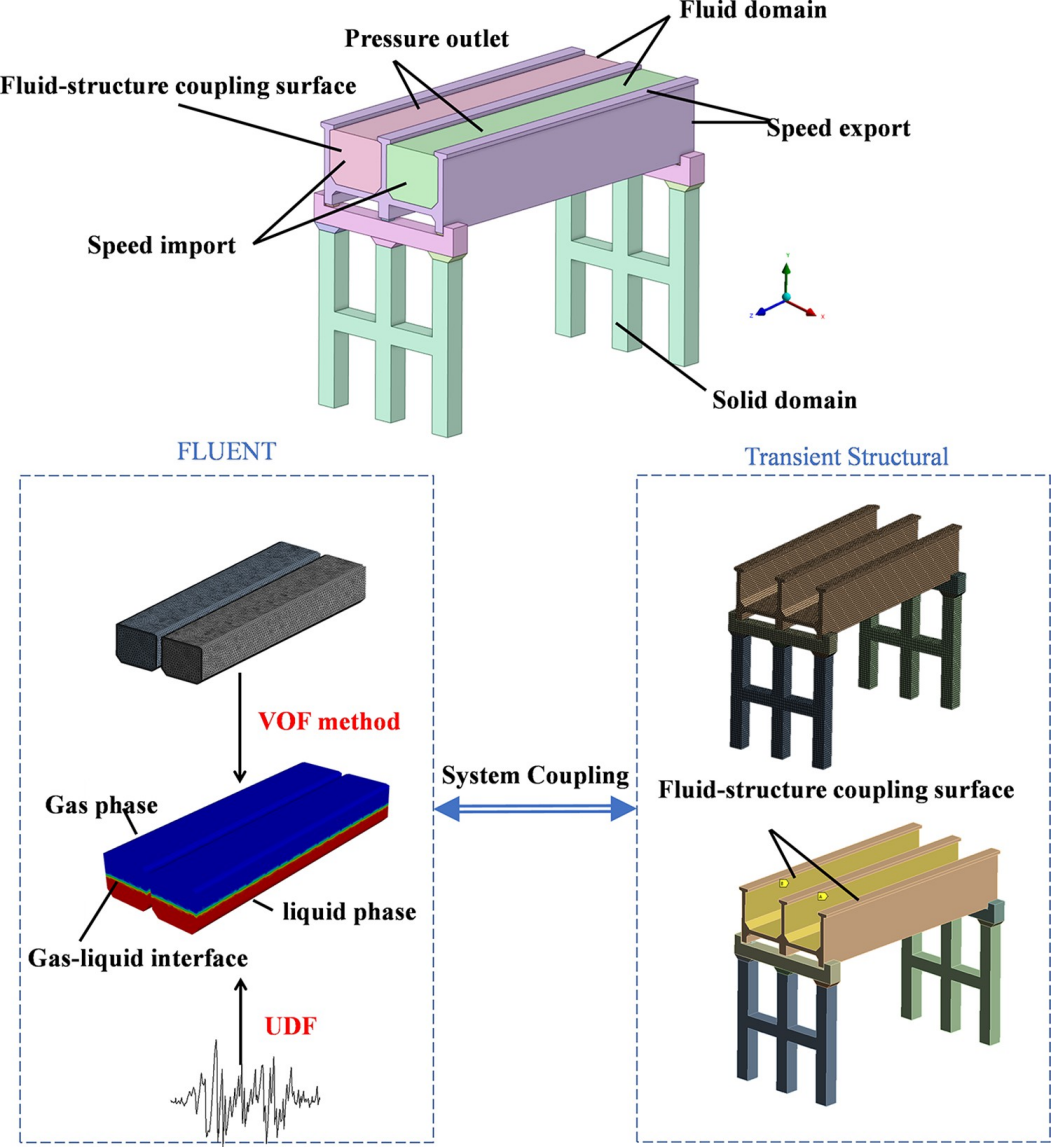
Fluid-structure interaction model.

The calculation model diagram is shown in the Fig 3, and the data of the fluid-structure interaction surface is exchanged through System Coupling. The pressure-based transient model is selected for the solver type in Fluent, and the VOF method is used to divide the entire fluid domain into the gas phase and liquid phase [22, 23], while considering the surface tension coefficient of the liquid (Among them, the gas phase is filled with air and the liquid phase is filled with water). The calculation model uses K-epsilon in the turbulence model and uses the standard wall function. Using the momentum source method, UDFs are used to apply acceleration in the lower x direction to the entire fluid domain, and the surface working air pressure of the domain is one standard atmospheric pressure. The solid domain does not consider the pile-soil action, does not consider the slip of the support and the superstructure, and applies seismic acceleration in the x direction. The specific 3D model is shown in the figure below. The trough body, pier columns and cover beams are all made of C30 concrete, and the specific material parameters are shown in Table 1. Assuming that the water is incompressible, the aqueduct walls are assumed to be rigid. However, to consider the water-aqueduct interaction, surface-to-surface hard contact with the body as 100% master and friction-less tangential behavior with finite sliding was assumed, which include nonlinear geometric effects such as large deformations and large rotations. Two-way coupling requires high computer performance, and the data does not converge due to grid changes and data transfer during the calculation process.

**Table 1:** Material properties.

Material	Density (kg/m3)	Elastic modulus (Pa)	Poisson’s ratio	Velocity (m/s)	Viscosity (kg/ (m*s))
**Liquid water**	998.2	-	-	14821	0.001
**Concrete**	2300	3e+10	0.3	-	-
**Air**	1.225	-	-	346.25	0.000018

### 2.3 Numerical simulation of working condition design

**Fig 4:**
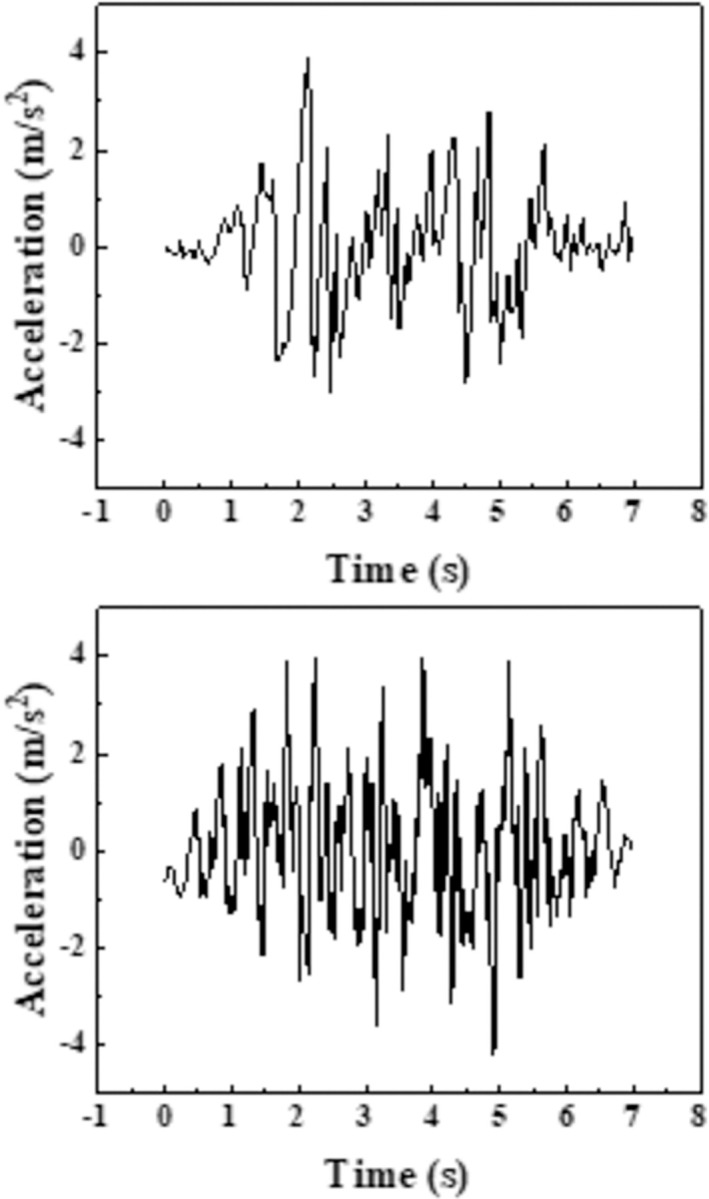
Schematic diagram of seismic waves. (a) EL wave. (b) artificial wave.

In order to study the dynamic response of aqueduct structure under earthquake, this paper selects seismic waves according to the site type and structural self-resonance characteristics specified in the "China Seismic Parameter Zoning Map". EL-centro waves were selected and an artificial wave was generated according to the geological data. Adjustment of the amplitude of the frequency and the specific value of the seismic waves were shown in the Fig 4. To further explore the influence of different water levels on the structure of the aqueduct under different earthquakes, the depth of the aqueduct is L, and a variety of numerical simulation conditions are set, as shown in the Table 2. (Note: The W-1 code refers to working condition one).

**Table 2:** The details of the working condition.

Working conditions	Water level	Seismic wave
**W-1**	0	EL-centro
**W-2**	0.3L	EL-centro
**W-3**	0.5L	EL-centro
**W-4**	0.7L	EL-centro
**W-5**	0	Artificial waves
**W-6**	0.3L	Artificial waves
**W-7**	0.5L	Artificial waves
**W-8**	0.7L	Artificial waves

### 2.4 Double trough aqueduct shaker test

The shaking table test of structural models is an important means to study the seismic performance, dynamic characteristics, seismic response, failure characteristics of structures, and the verification of calculation models for engineering structures.

**Fig 5:**
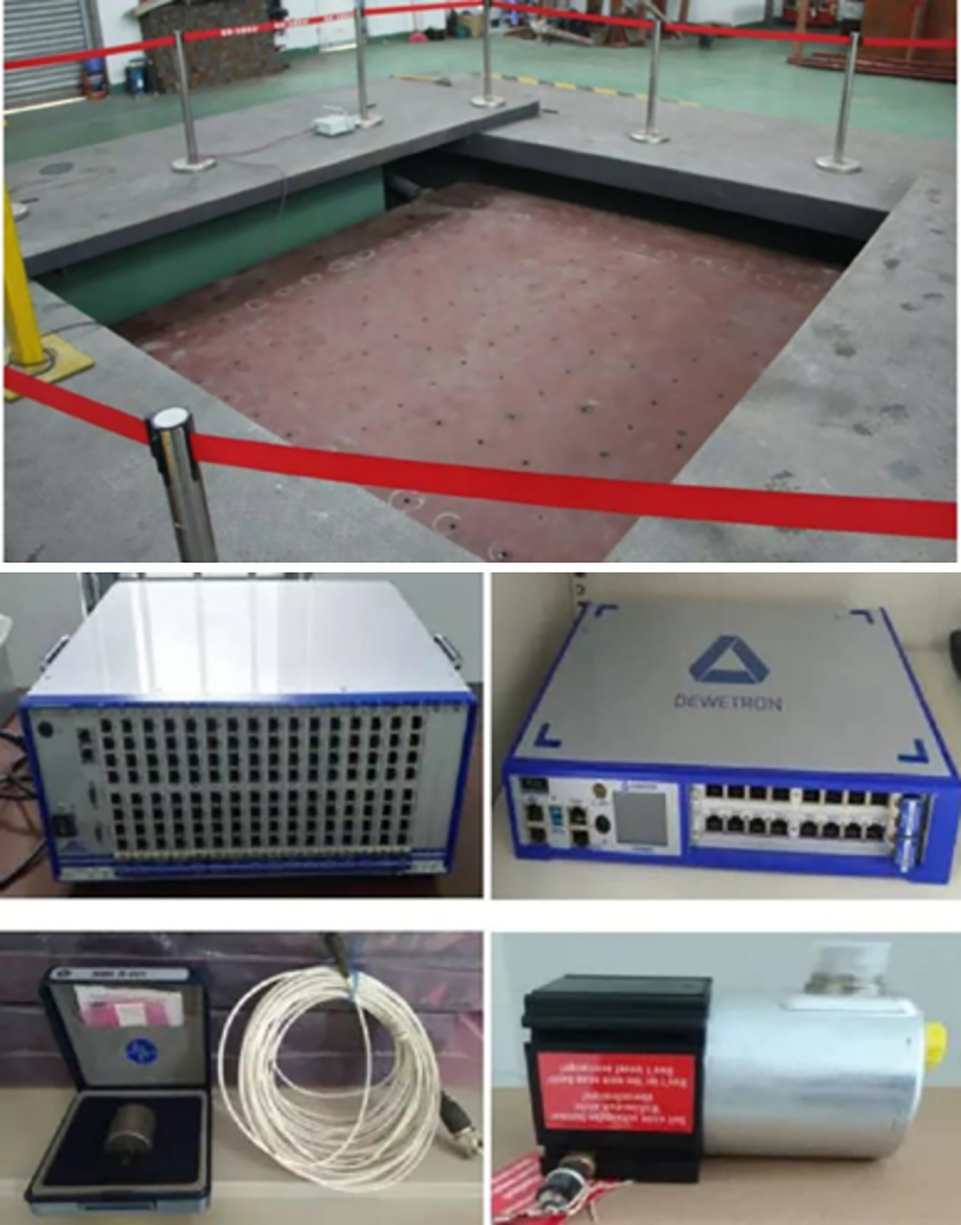
Shaking table and its test equipment.

The test was carried out in the seismic simulation shaker laboratory of South China University of Technology, the shaking table is a 4m×4m three-way six-degree-of-freedom seismic simulation shaker, the maximum horizontal acceleration is 1g, the vertical acceleration is 2g, which can accurately simulate ground motion. The data acquisition system was tested by the Austrian Dewetron data acquisition system, the American PCB accelerometer and the ME’scopeVES modal analysis system, and the specific equipment is shown in the Fig 5.

Combined with the size of the shaker, the test aqueduct is scaled down according to the original structure ratio 1:6. Among them, the aqueduct model adopts the ignoring gravity model, that is, the simulation of gravitational acceleration is not considered in the model design, that is, the similar requirements of Sg = 1 are ignored, so that SE, Sl and Sρ can be freely selected independently. Sl, SE, and Sα are taken as the basic similarity coefficients, and the specific similarity relationship of this model is shown in the Table 3.

**Table 3:** Aqueduct scale model structural similarity (ignoring gravity model).

	physical quantity	Similar metrics	numeric value	remark
**Geometric properties**	Geometric Dimensions *l*	S_*l*_	1/6	Size control parameters
	Line displacement *u*	S_*u*_ = S_*l*_	1/6	
	Angular Displacement *β*	S_*β*_ = S_*σ*_/ S_*E*_	1	
**Material properties**	Elastic modulus *E*	S_*E*_	1	Material control parameters
	Quality *m*	S_*m*_ = S_*E*_ S_*l*_^2^/ S_*α*_	1/36	
	Equivalent mass density *ρ*_*eq*_	S_*ρ* eq_ *=* S_*m*_/ S_*l*_^3^	6	
	Stress *σ*	S_*σ*_ = S_*E*_	1	
	Strain *Ɛ*	S_*Ɛ*_ = S_*σ*_/ S_*E*_	1	
**Load characteristics**	Concentration *F*	S_*F*_ = S_*σ*_S_*l*_^2^	1/36	
	Line loads *p*	S_*p*_ = S_*E*_ S_*l*_	1/6	
**Dynamic characteristics**	Acceleration *α*	S_*α*_	1	Test control parameters
	Torque *M*	S_*M*_ = S_*E*_ S_*l*_^3^	1/216	
	Cycle *T*	S_*T*_ = (S_*l*_ / S_*α*_)^1/2^	1/2.45	
	Vibration frequency *f*	S_*f*_ = (S_*l*_ / S_*α*_)^-1/2^	2.45	
	Dynamic reaction Speed *v*	S_*v*_ = (S_*l*_ S_*α*_)^1/2^	1/2.45	
	Damping *c*	S_*c*_ = S_*l*_^1.5^ S_*α*_^-1/2^ S_*σ*_	1/14.70	

**Fig 6:**
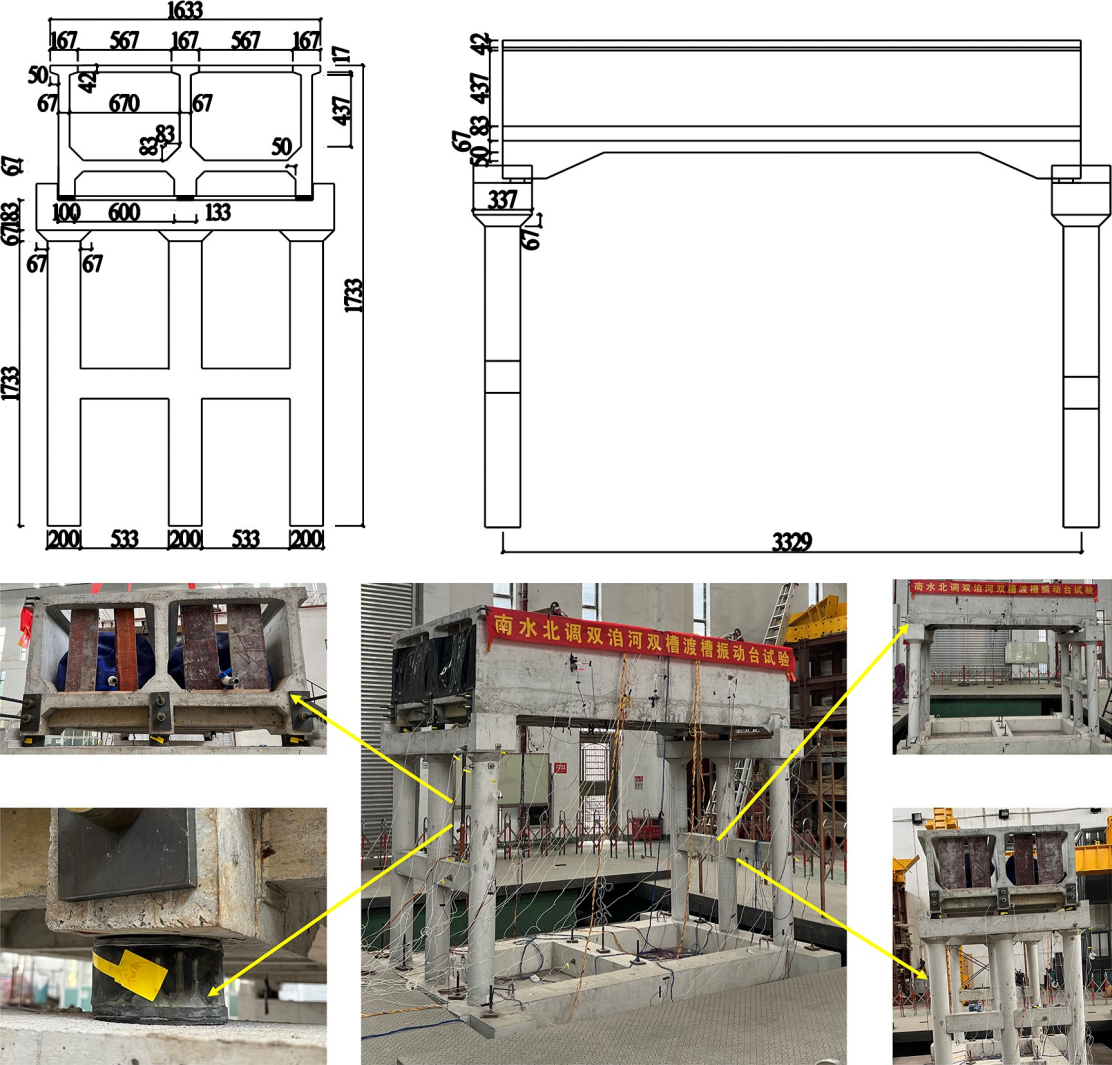
Test model details and test models.

The model takes a single span, and the model design diagram is given according to the experimental similarity ratio, as shown in the Fig 6.

### 2.5 Arrangement of sensors

In the test, to obtain the acceleration response, displacement reaction, hydrodynamic pressure reaction and strain reaction of the aqueduct structure model, accelerometer, displacement meter, hydrodynamic pressure gauge and strain gauge are arranged on the model. During the test, the data acquisition and processing system automatically collects the reaction data of the model structure under different table excitation.

**Fig 7:**
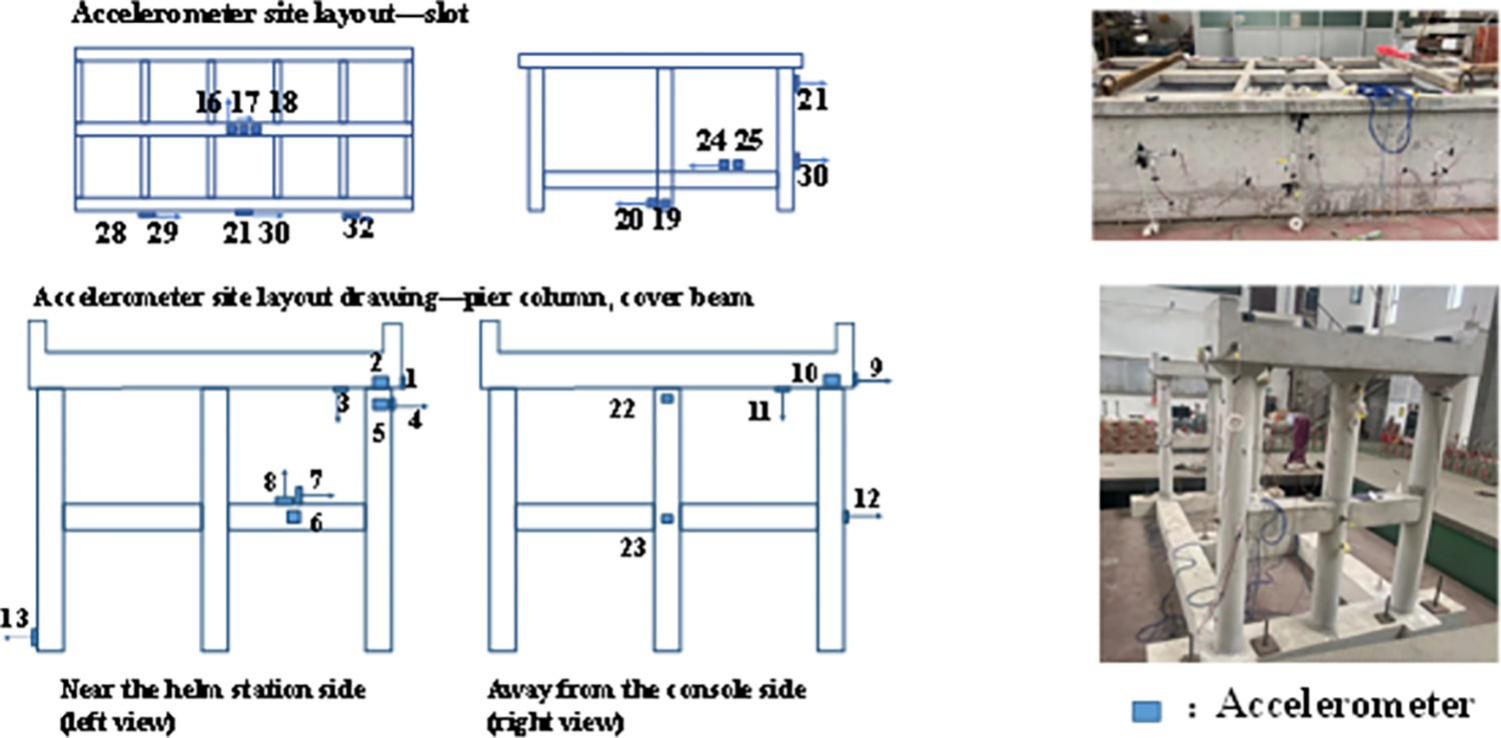
Aqueduct model sensor layout drawing.

According to the collected seismic response data and the observed damage of the model structure, the response of the prototype structure under the action of the earthquake and its comprehensive seismic performance can be analyzed and inferred. Fig 7 shows the sensor layout on the aqueduct structure model.

### 2.6 Analysis of test results

**Fig 8:**
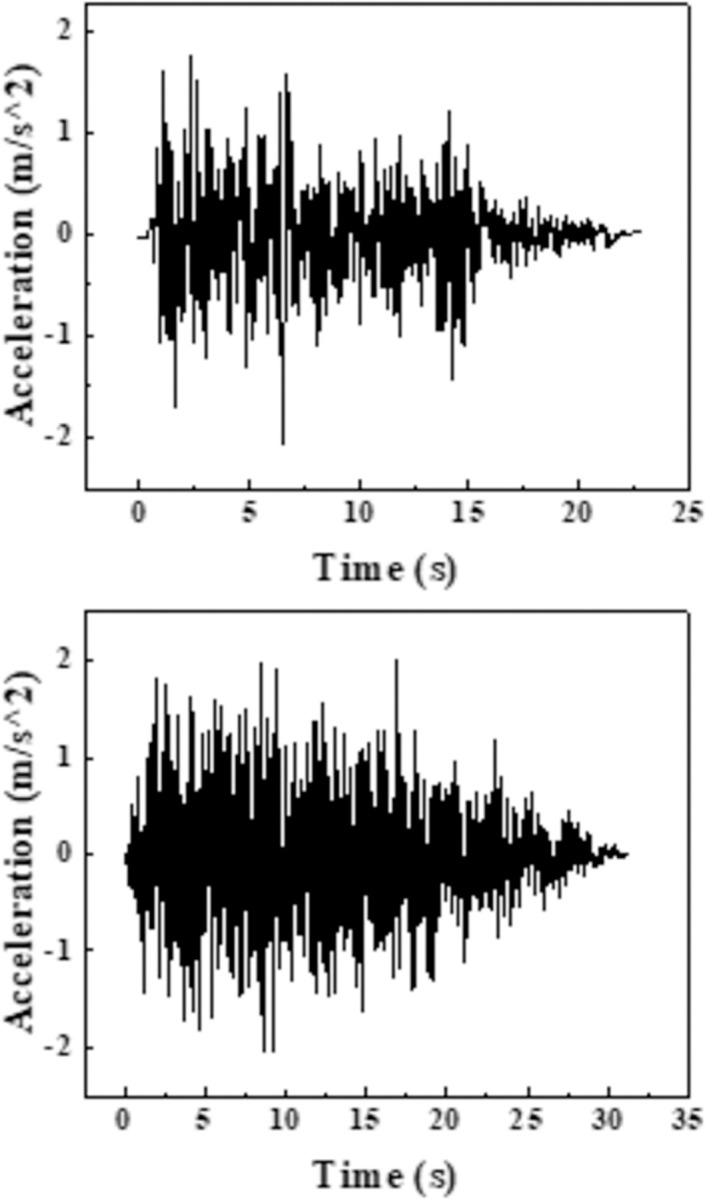
Seismic waves selected for the test.

For the three different water level conditions of empty trough, 3/L water depth and 2/3L water depth, the seismic wave input takes 0.2g, 0.4g El-Centro wave and artificial wave, and frequency modulation amplitude adjustment, the seismic wave time history diagram is shown in the Fig 5. The results of this paper only show the working conditions of the aqueduct structure in the peak acceleration of 0.2g El Centro wave and artificial wave, as shown in the Fig 8.

**Fig 9:**
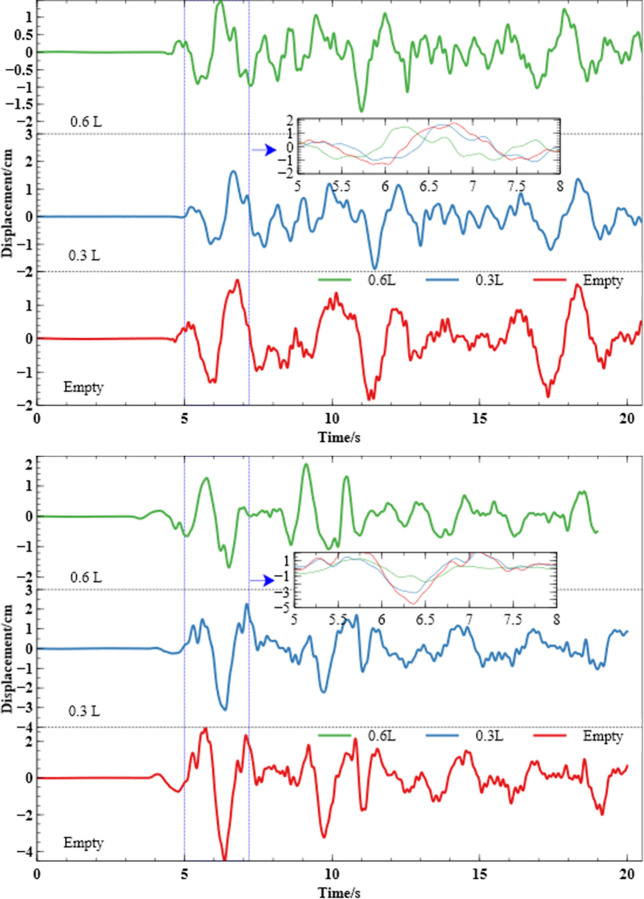
The displacement of the aqueduct at different water depths corresponds.

The mid-displacement of the aqueduct span at different water levels is shown in the Fig 9(A). At the peak displacement, the mid-span displacement value of the empty trough was 4.57cm, and the shock absorption rate at water level 0.3L and 0.6L was 31.7% (displacement value: 3.12cm) and 63.4% (displacement value: 1.67cm), respectively, indicating that the fluid in the aqueduct could weaken the effect of the aqueduct under the action of an earthquake. The displacement amplitude of the empty tank at 7s was 2.32cm, the amplitude of the water level 0.3L displacement was 2.25cm, the displacement of the empty trough at 9s was 1.78cm, and the amplitude of the water level 0.6L displacement was 1.71cm, showing that the fluid did not play a shock absorption role. It should be noted however that the presence of fluid at 18s enhances the seismic response of the structure. In general, the fluid-structure interaction effect of the aqueduct plays a role in damping the shock most of the time. The greater the damping rate the more the increase of the water levels.

The mid-span displacement of the aqueduct under the action of artificial waves is shown in the Fig 9(B), and the peak displacement of the empty trough is -1.83cm. The displacement value of the aqueduct span at 0.3L was -1.89cm, which was manifested as increasing the displacement response of the structure, and the displacement value at 0.6L was -1.7cm, which showed that the displacement response of the structure was weakened. With the increase of water level, the seismic response to minimize appeared around 17s, when the displacement value of the empty trough was -1.75cm, the shock absorption rate at 0.3L water level was 30.5% (displacement value: -1.21cm), and the shock absorption rate at 0.6L water level was 41.1% (displacement value: -1.02cm). At most peaks, with the increase of water level, the stronger the effect of the liquid on the aqueduct, the better the shock absorption effect, and the shaking effect of the liquid at the individual peaks increases the effect of the structure under the action of earthquakes.

### 2.7 Numerical simulation and its validation

**Fig 10:**
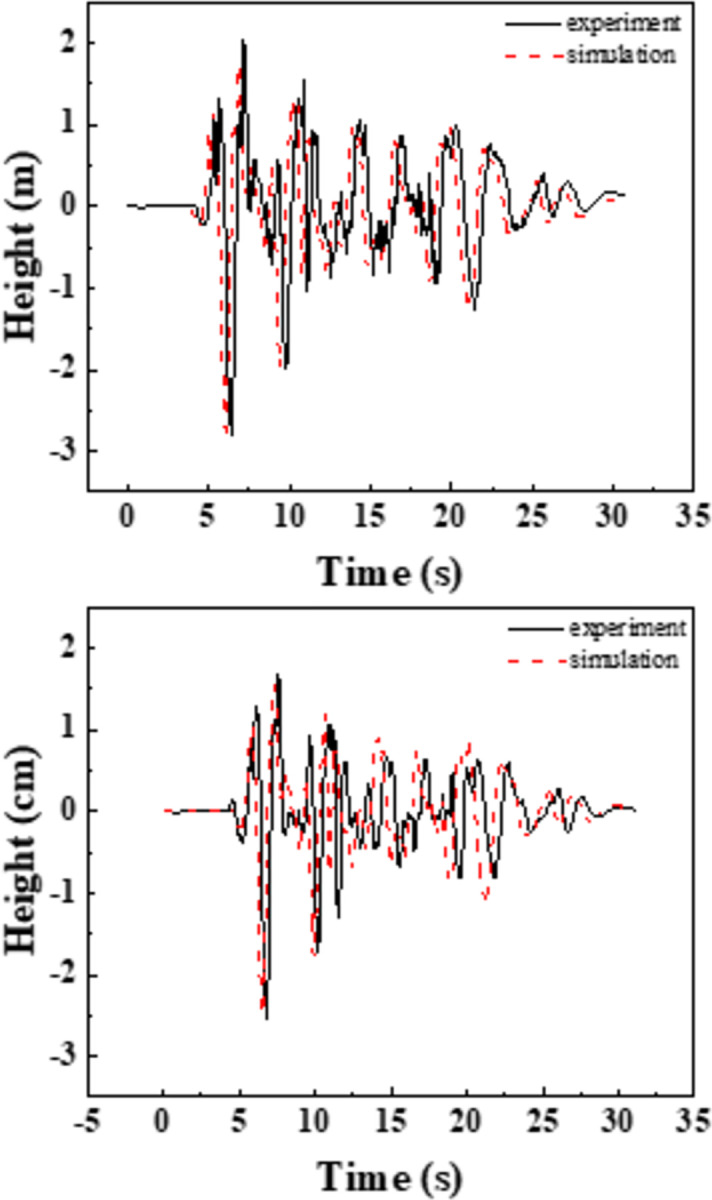
Comparison of test and simulation results.

In order to verify the accuracy of the finite element model, the aqueduct scale test model is established by the bidirectional coupling method described above, simulating the aqueduct test water depth of 1/3L, 2/3L, and the lateral excitation under 0.2g El-Centro wave, Fig 10 shows the results of the aqueduct span displacement in the finite element and the test respectively. The test results and the numerical results are in good agreement, their overall trend changes are consistent, and the peak height is within the allowable error range.

## 3. Finite element result analysis

### 3.1 Solid domain displacement analysis

**Fig 11:**
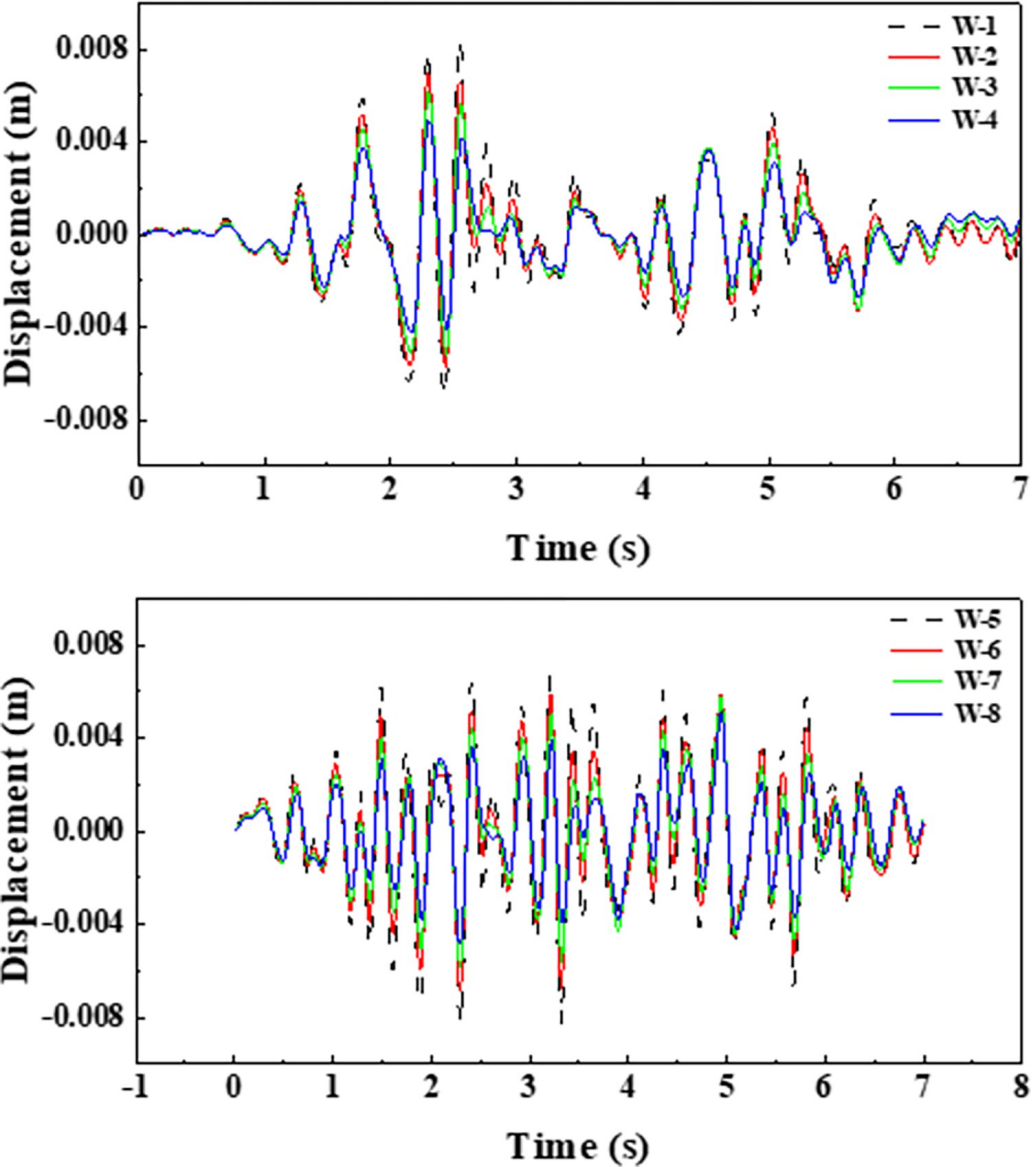
The mid-displacement of the aqueduct span under different seismic waves. (a) Seismic response under EL waves. (b) Seismic response under artificial waves.

The trans-mid-displacement response of aqueducts with different water levels under different seismic waves is shown in the Fig 11. The change in water level height has a significant effect on the displacement in the trough direction. The Fig 11(A) shows that around 1.8s, the displacement amplitude of the empty slot is 5.87mm, and the displacement amplitude of working conditions two ~ four is 88.2% (5.81mm), 78.2% (4.59mm) and 64.1% (3.76mm) of the empty groove, respectively; around 2.55s, the displacement of the aqueduct with water and the aqueduct without water reaches the peak at the same time, the displacement amplitude of the empty trough is 8.3mm, and the displacement amplitude of the working condition 2~4 is 79.2% (6.57mm) of the empty groove 68.0% (5.64) and 49.6% (4.12), and around 5.5s, the displacement amplitude of the empty slot is 1.21mm, and the displacement amplitude of working conditions two ~ four is 137.2% (1.66mm), 168.6% (2.04mm) and 171.1% (2.07mm) of the empty groove, respectively. It shows that when the liquid is around 1.8s and 2.5s, the movement direction of the liquid and the structure is opposite, which can play a role in TLD shock absorption to a certain extent; Around 5.5 seconds, the liquid moves in the same direction as the structure, increasing the structure’s displacement response. Before 4s, the displacement amplitude of anhydrous aqueducts and water-based aqueducts with different water levels reached the peak at almost the same time point, and after 4s, such as 4.28s, 5s, and 5.84s, there was a certain degree of lag, indicating that with the increase of water level, the hysteresis of the peak displacement of the aqueduct was also more obvious. As shown in the Fig 11(B), in the more peak output, the presence of liquid plays a significant TLD effect, artificial waves reach the first maximum peak at 2.3s, at this time the displacement amplitude of the empty slot is 8.26mm, and the displacement amplitude of working conditions six ~ eight is 82.8% (6.84mm), 70.2% (5.80mm) and 59.0% (4.87mm) of the empty groove, respectively. Around 2.1s, the artificial wave acceleration decreases sharply, at this time the displacement of the empty slot span drops to 1.1mm, the fourth displacement of the working condition is reduced from the previous peak to 2.34mm, and the working conditions seven and eight increase to 2.99mm and 3.14mm respectively due to the large mass of the liquid, at this time the presence of liquid increases the displacement of the structure.

### 3.2 Fluid domain water level analysis

**Fig 12:**
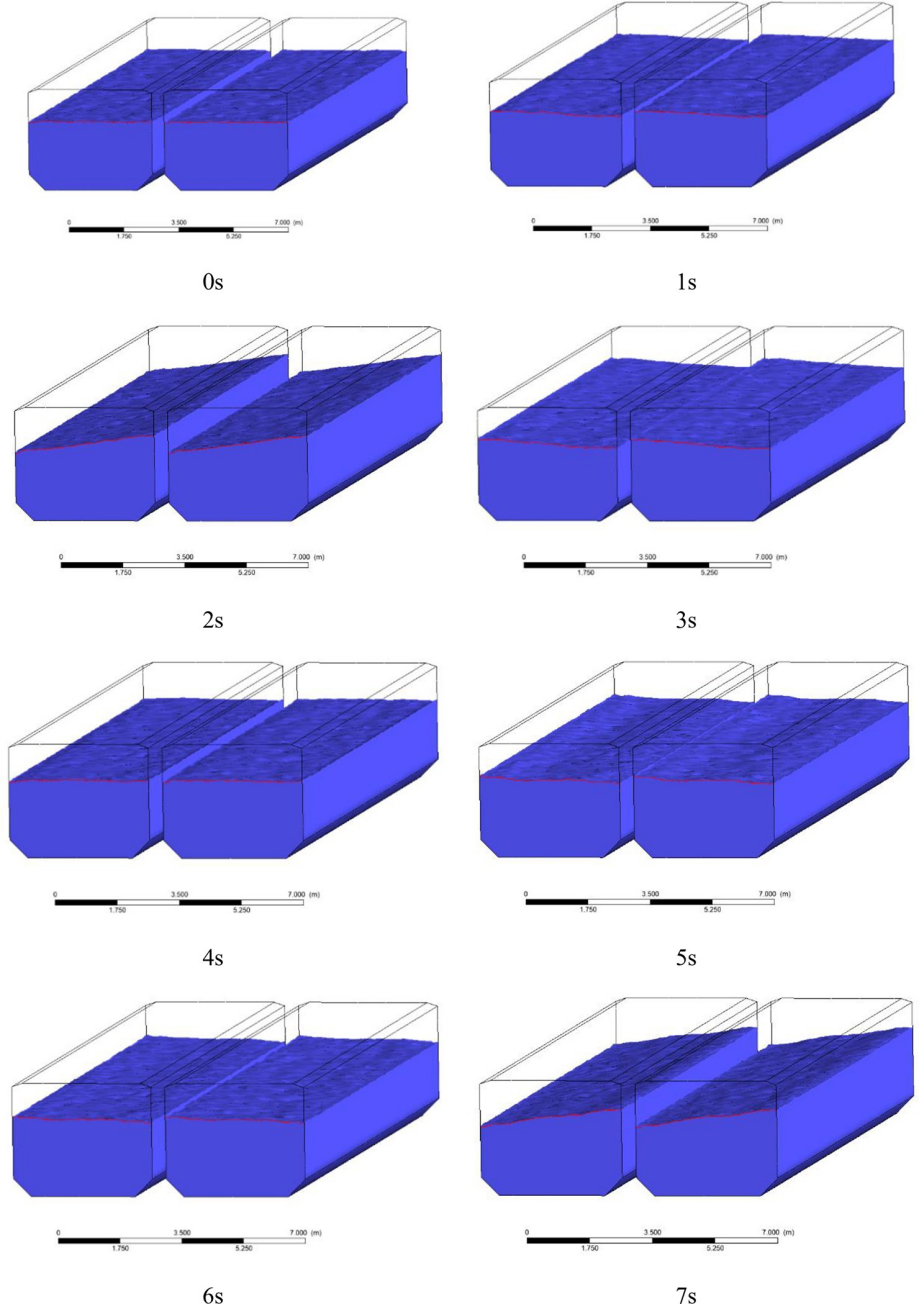
Water level changes within 0~7s.

As shown in the Fig 12, taking working condition 8 as an example to show the water level change under the action of earthquake, the definition of the trough surface is left wall (LLF), left groove right wall (LRF), right groove left wall (RLF) and right groove right wall (RRF) from left to right.

**Fig 13:**
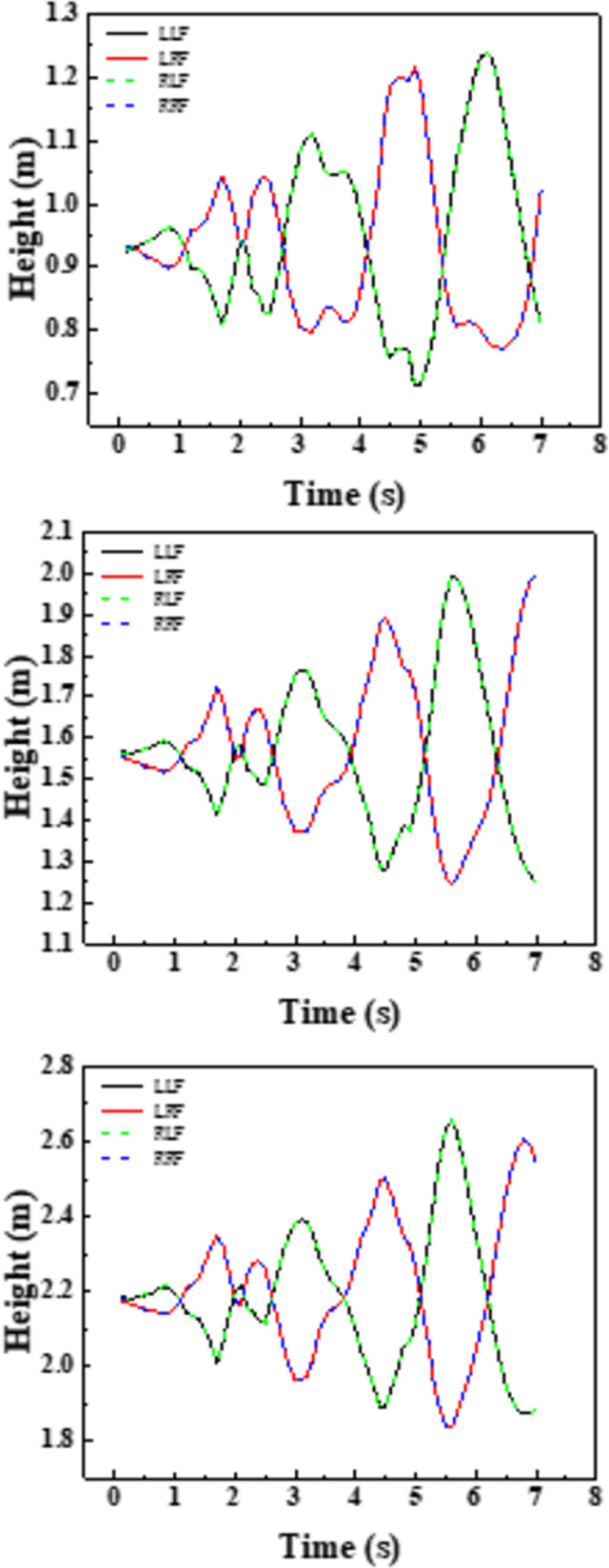
Water level change under working conditions 2~4.

**Fig 14:**
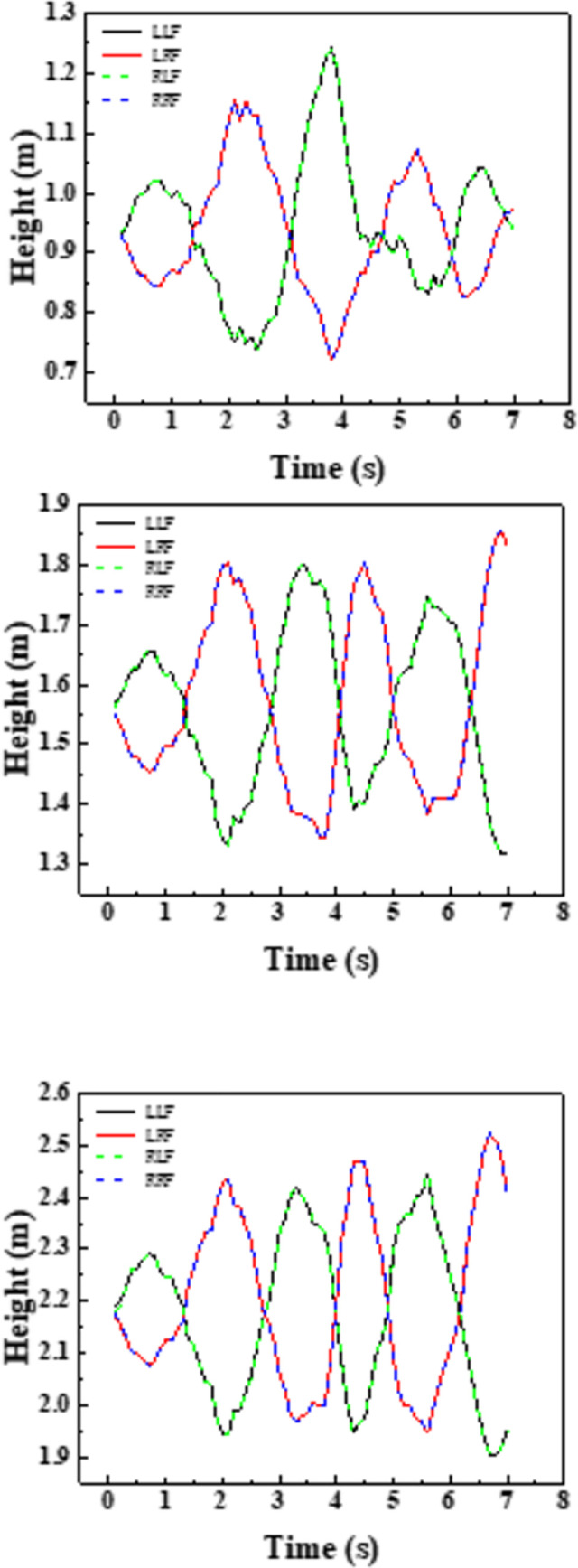
Water level change under working conditions 6~8.

Fig 13 shows the water level change of working conditions two ~ four, the water surface fluctuation of the left groove and the right trough shows consistency, under the action of the earthquake has experienced 4 processes from the peak to the trough, with the increase of the water level, the position and cycle of the peak trough have not changed significantly, and the peak connection is shown in the figure. The Fig 4(A) shows at 2.1 s, the peak of the seismic wave reaches its maximum, and the amplitude of the cross-mid-displacement of the structure also reaches its maximum, which on the contrary, is not at the peak in the fluid domain. The peak of the fluid domain is formed around 6s at the end of the seismic wave, which is out of sync with the peak of the seismic wave and has obvious hysteresis. Fig 14 shows the water level change of each tank surface under working conditions 4~6, which is different from the working conditions under natural waves. The different working conditions under artificial waves have experienced three processes from crest to trough. Compared with natural waves, artificial waves have more peaks and more disguised forms. In contrast, the aqueduct solid domain shows multi-peak and multi-disguised results, but the liquid domain results are exactly the opposite, indicating the non-synchronization of liquid and peaks.

**Fig 15:**
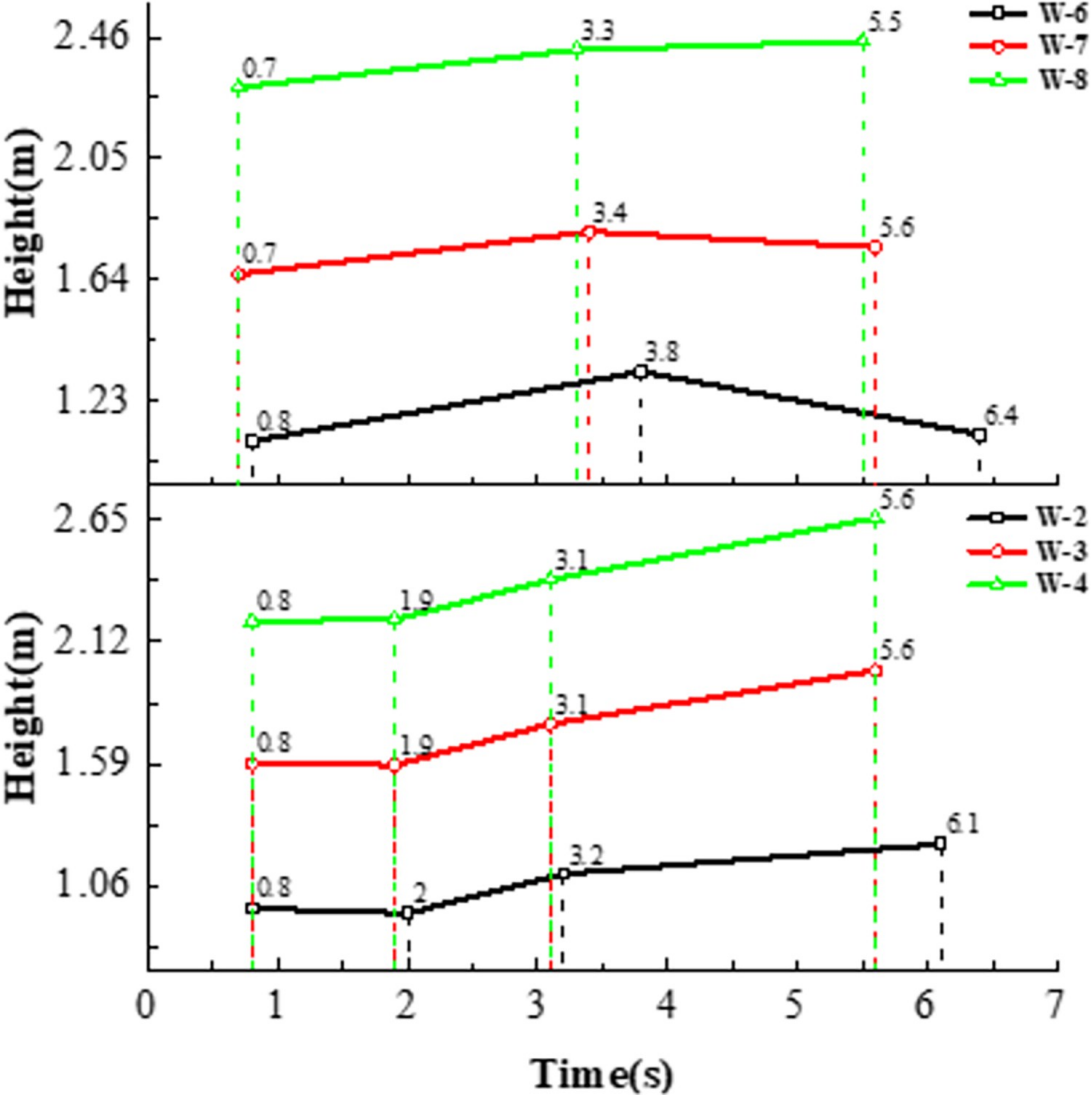
Crest changes at different water levels.

The Fig 15 shows the connection of the water level peak point on the left side (LLF) of the left groove under each working condition, including the liquid level change of the left groove and the right groove under the action of seismic earthquake, activity. Taking working conditions 6~8 as an example, the time for working condition 6 to reach the first wave peak is 0.8s, working condition 7 and working condition 8 are 12.5% (0.1s); the time for working condition 6 to reach the second wave peak is 3.8s, and the time to reach the second wave peak in working condition 7 and working condition 8 is 10.5% (0.4s) and 13.2% ahead of 13.2% respectively (0.5s); the time for the third wave of condition 6 is 6.4s, and the time for working condition 7 and working condition 8 is 12.5% (0.8s) and 14.1% (0.9s) ahead of time, respectively. As the water level increases, the more the energy absorbed by the same seismic use causing the time for the liquid level to reach its peak becoming advanced. Performance is more obvious as the action time increases.

### 3.3 TLD mechanism analysis

According to the principle of conservation of energy, TLD reduces the vibration of the structure by converting the kinetic energy of the structure into the sloshing energy of the water body. The energy consumption of dangling water bodies in water aqueducts is essentially achieved through internal vortices [24]. The presence of a wall has a significant effect on the flow field. To explore the damping excitation of TLD in the aqueduct, the dimensionless free liquid surface wave height, dimensionless horizontal control force and wet mode were analyzed.

#### 3.3.1 Free liquid level shaking amplitude

Definition: $\eta^{\prime }=\frac{\eta }{h}$. where *η* is the height of the free liquid level at the side wall of the tank at a certain time, and *h* is the height of the liquid in the tank. *η*′ is the amplitude of free liquid surface shaking.

**Fig 16:**
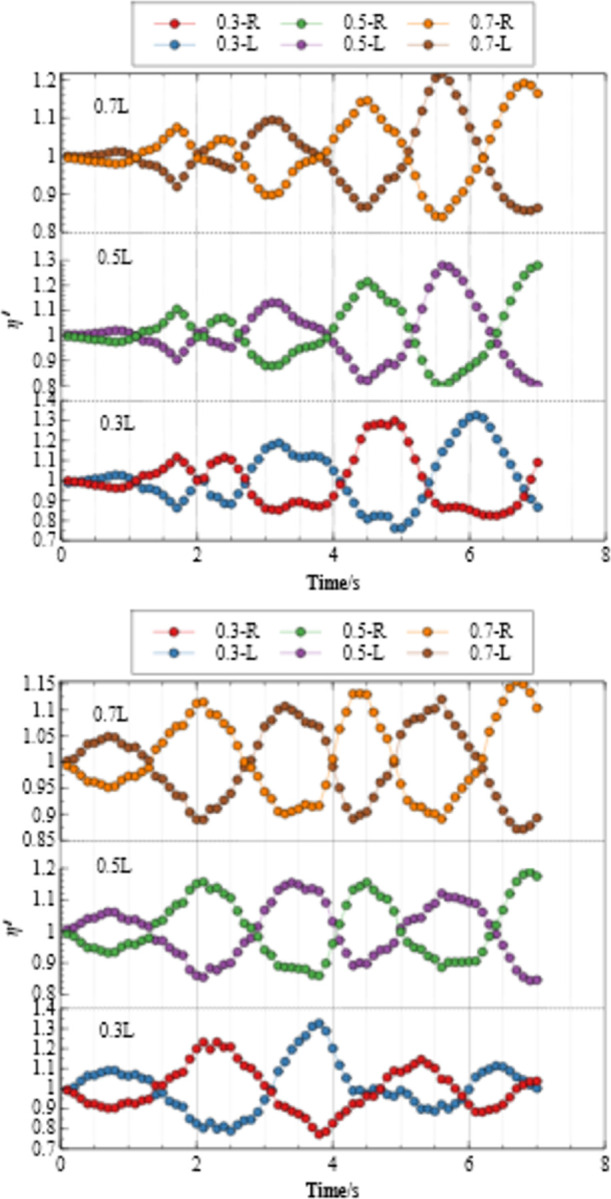
The amplitude of liquid sloshing under different working conditions. (a) Details of EL waves. (b) Details of artificial waves.

The Fig 16 shows the water level change diagram of the left groove left and right walls under different working conditions. As shown in the Fig 16(A), before 3s, different water levels reach the peak at the same time. Over time, under the 0.3L water level condition, the left wall of the left groove reaches a peak at 4.8s, while the 0.5L water level and 0.7L water level reach a peak at 4.5s and 4.4s, respectively; The 0.3L water level reached the next peak at 6.1, while the 0.5L water level and 0.7L water level reached peak in 5.5s and 5.4s, respectively. The results show that as the water level increases, the smaller the time from the peak to the trough (negatively correlating with the water level). When the liquid water level is at a low value, the sidewall water level changes greatly. With the increase of water level, the shaking amplitude decreases, and the water level change is negatively nonlinear with the shaking amplitude. A more obvious pattern also appears in Fig 16(B), where the amplitude of the surface shaking of the left and right wall water levels is mirrored. Due to the large and concentrated peaks of artificial seismic waves, the amplitude change under 0.3L water level reaches the maximum when it is close to 4s, which is much greater than that of other working conditions. In the period of 6~7s, the peak occurrence time under different working conditions shifted forward with the increase of water level, reflecting the strong nonlinear process in the fluid-structure interaction process.

#### 3.3.2 Hydrodynamic pressure

**Fig 17:**
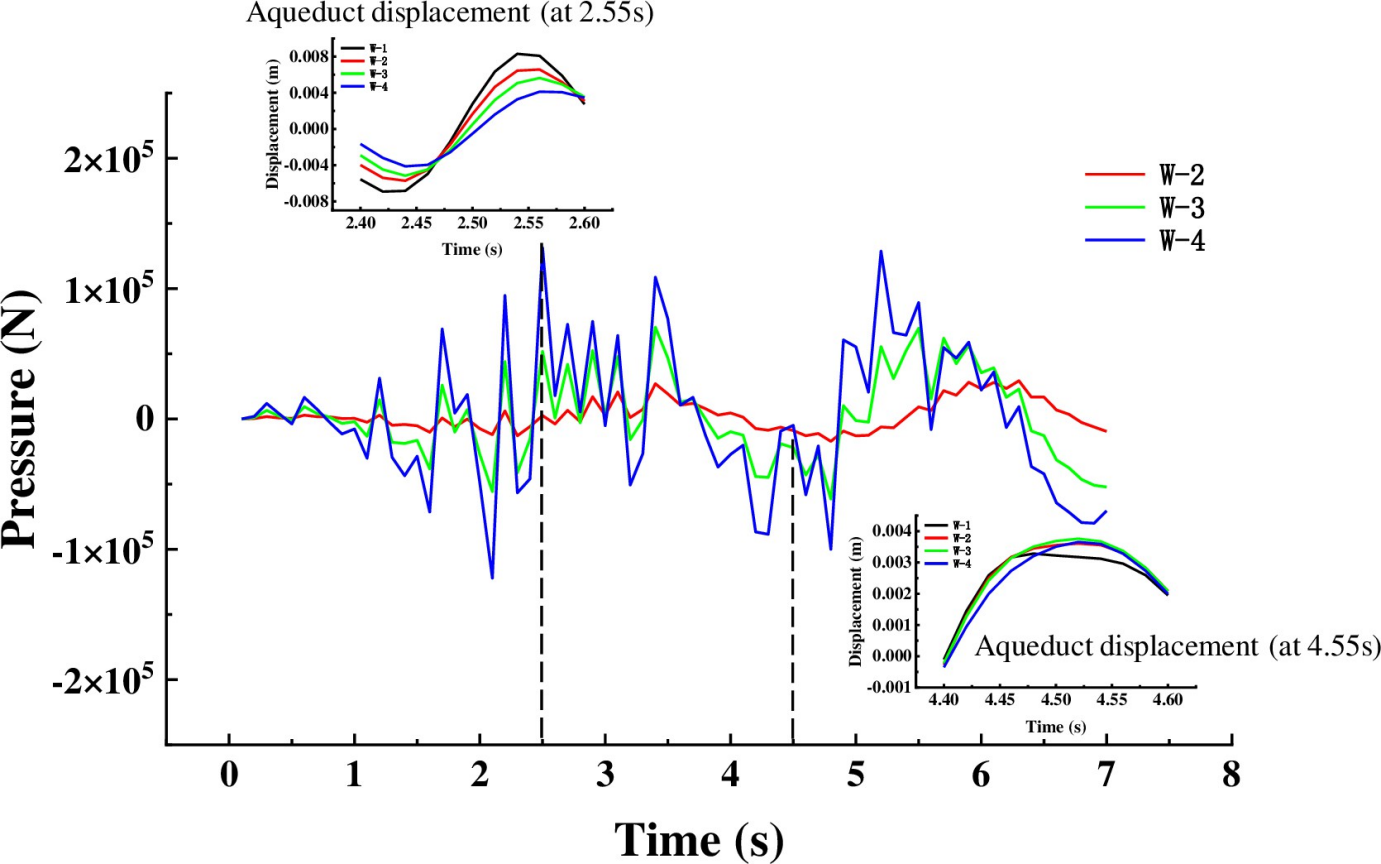
Horizontal force curves of different water levels under EL waves.

It is well known that TLD can use the hydrodynamic pressure generated during liquid shaking to provide shock absorption, and it is concluded above that the liquid domain changes in the two tanks are consistent. Taking the left groove as the research object, the collected hydrodynamic pressure value curve is shown in the Fig 17. When the amplitude of the middle displacement of the aqueduct span reaches the peak, the hydrodynamic pressure of the corresponding aqueduct wall also reaches the peak. When the hydrodynamic pressure value is 0, the amplitude of the corresponding aqueduct span displacement is close to equal at different water levels, indicating that the aqueduct structure does not receive the horizontal force of the liquid in the horizontal direction at this time, and does not play a shock absorption role at this time. Around 2.5s, as the water level increases, the greater the hydrodynamic pressure value, the smaller the mid-displacement of the aqueduct span. At 3.2s, the hydrodynamic pressure peaks in the opposite direction, at which time the trans-center displacement also increases with the increase of the water level, and the shaking of the liquid increases the response of the structure under the action of earthquake.

**Fig 18:**
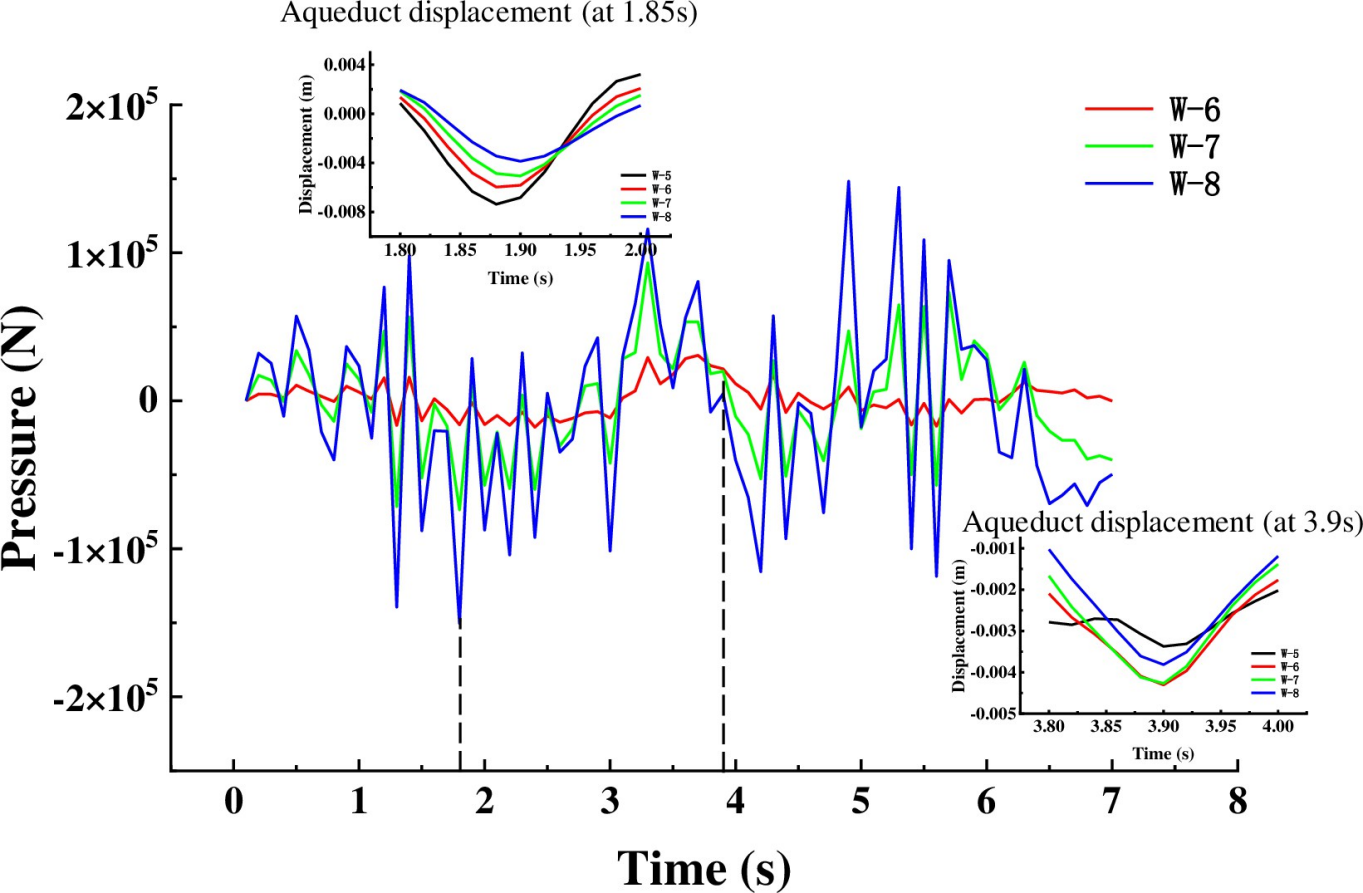
Horizontal force curves of different water levels under artificial waves.

The Fig 18 shows the hydrodynamic pressure curve under the artificial wave condition, the peak value appears at 1.85s, when the mid-displacement of the aqueduct span under the artificial wave is the smallest, and the TLD effect enhances with the increase of water level. At 3.9s, the horizontal control force of working condition 7 is equal to working condition 6, and both are less than working condition 8, so the displacement amplitude of working condition 6 is equal to that of working condition 7 in Fig 10(B) at 3.9s, and both are greater than working condition 8. The above results show that the liquid in the tank can play a role in shock absorption in most cases, and will increase the seismic effect of the structure in a few times.

## 4. Water-filled mode of the aqueduct

**Fig 19:**
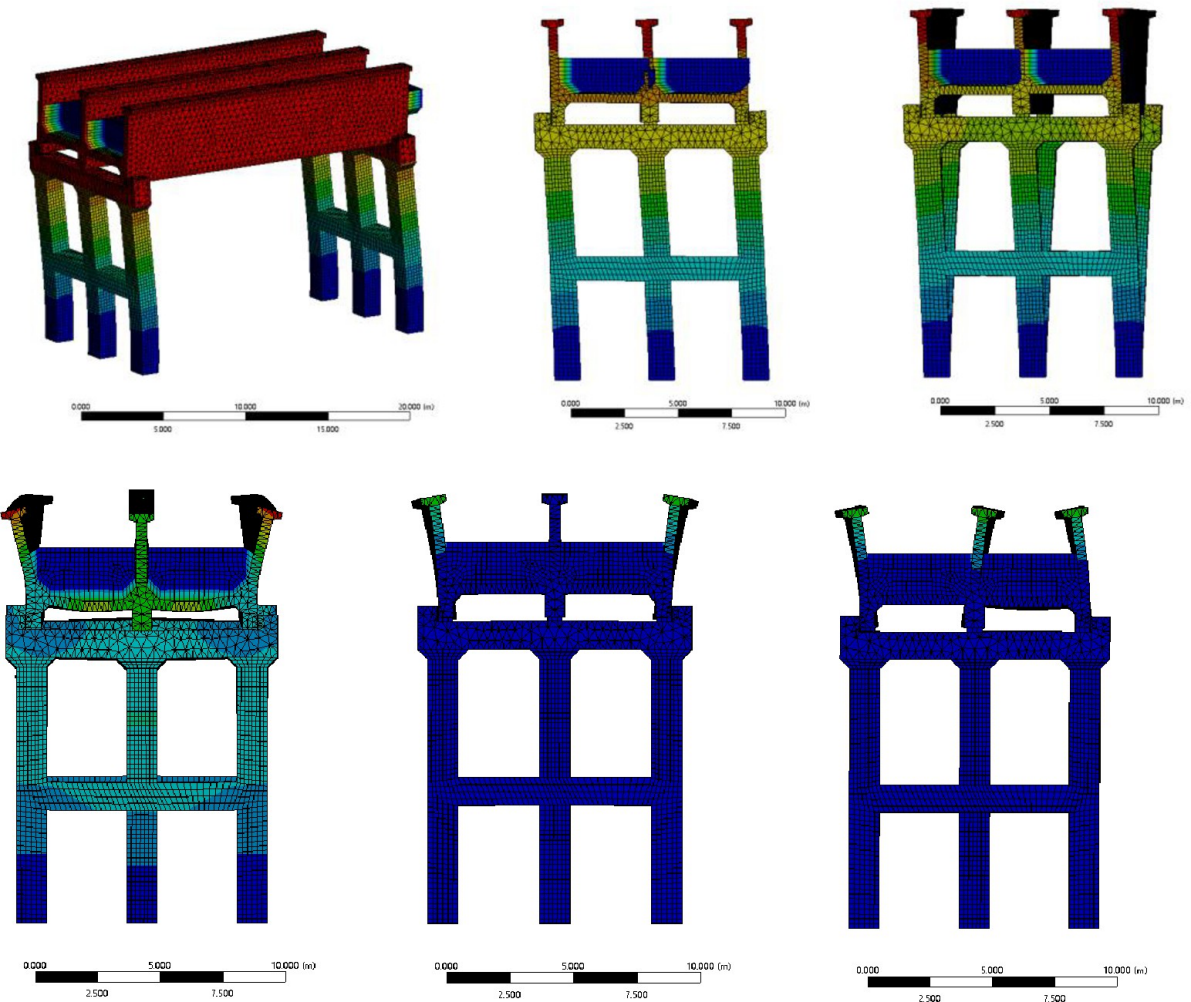
Diagram of the first sixth-order mode shape of the aqueduct.

The modal analysis of the aqueduct structure is the basis for the seismic design and seismic response analysis of the aqueduct, mainly including the self-resonance frequency and main mode shape [25]. The self-resonance frequency can not only characterize the rigidity of the aqueduct structure, but also be used as the basis for determining whether the aqueduct structure can resonate. The main mode shape is closely related to the state of structural dynamic reaction. The dynamic characteristics analysis of fluid-structure interaction structure needs to consider the effect of water on the aqueduct, involving the simulation and simplification of the action of the water body, Yang Guodong et al. [26] show that for the calculation of the low self-resonance frequency of an immersed cylindrical shell, the dynamic load of the fluid on the structure in the fluid-structure interaction model and the acoustic-solid interaction model can be degraded into a unified form, which is consistent in solving the low-frequency self-resonance characteristics. With the former, a liquid-containing aqueduct acoustic-solid interaction model is established. The sound velocity of liquid water is defined as 1482.1m/s, and the characteristics of the first 6th order mode shape of the aqueduct are shown in the Fig 19 (due to the small difference in the mode shape of the aqueduct at different water levels, take the half-water level as an example). The first-order mode and the second-order mode are mainly based on the overall bending of the aqueduct structure, the third-order mode is manifested as the twisting of the trough, and the fourth-order, fifth-order and sixth-order modes are manifested as different degrees of bending and twisting of the trough wall with the change of water level. The details of the frequency values and mode shape shapes corresponding to the modes of each order are shown in Table 4.

**Table 4:** Self-oscillating frequency and mode shape at different water levels.

Modal	Empty	0.3L	0.5L	0.7L	Mode shape
**1**	2.1757	2.1604	2.1605	1.8365	Transverse bending
**2**	4.5188	4.0059	3.7859	3.4924	Longitudinal bending
**3**	6.2392	5.6256	5.37	5.0776	Longitudinal torsion
**4**	11.534	10.8922	10.188	8.7422	Groove wall torsion
**5**	12.308	11.9483	11.136	9.601	The groove walls are curved
**6**	14.438	12.659	11.716	9.9192	The groove walls are curved

**Fig 20:**
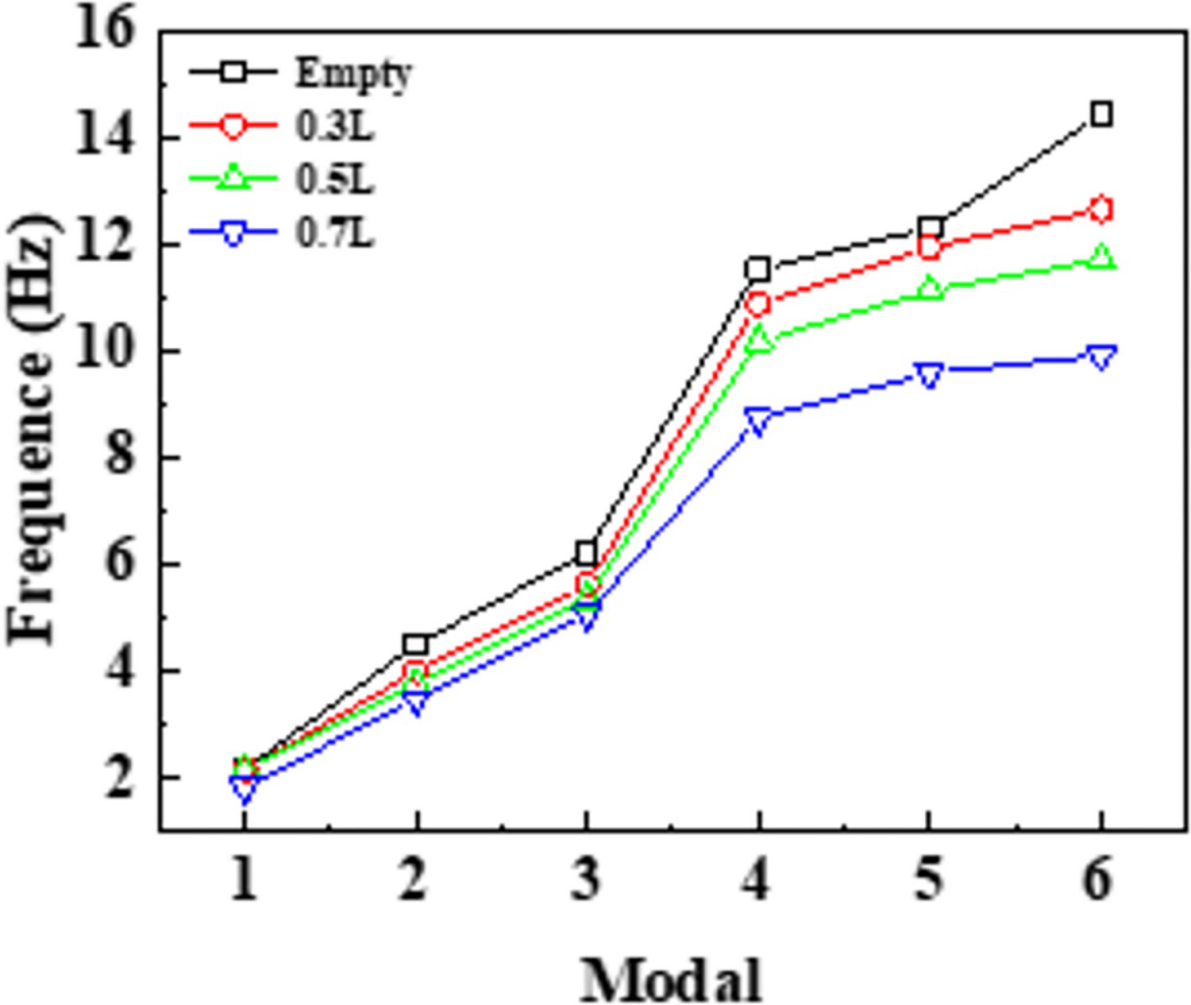
The self-resonating frequency of the aqueduct at different water levels.

As shown in the Fig 20, the excitation frequency generated by the aqueduct structure is mainly distributed in the low-frequency region, meaning the natural frequency of the structure is the largest when the trough is empty, and the existence of the liquid domain weakens the self-resonance frequency of the overall structure. As the water level rises to 0.7L, the local position of the high-order mode shape of the aqueduct structure changes significantly, resulting in a significant decrease in the natural frequency. On the whole, with the increase in water level, the modal coefficient of each order decreases and the natural frequency decreases.

## 5. Conclusion

In this paper, the seismic analysis of the double-trough aqueduct under the action of seismic action is studied, utilising a three-dimensional model of the double-trough aqueduct. Considering fluid-structure interaction that was established from the perspective of natural wave and artificial wave, combined with the analysis of various water level conditions, the following conclusions are obtained:

(1) Although there is a certain liquid level difference between the right wall of the left channel and the left wall of the right channel in the double-channel aqueduct, it does not affect the water level change of the left channel and the right groove, and the response of the liquid domain of the left and right groove is consistent under the action of an earthquake.

(2) The presence of liquid in the aqueduct can significantly reduce the displacement of the structure under the action of an earthquake, and the maximum shock absorption rate in different water levels under EL wave is 50.4%, whilst the artificial wave is 41%. Under partial seismic response, the excitation effect of the structure under the earthquake will be increased, with the EL wave increase by about 71% and the artificial wave increase by about 34%. Although the shock absorption time of the liquid in the tank is much greater than the excitation time, special attention should be paid to this when considering the seismic design of the aqueduct.

(3) The peak water level in the liquid area of the tank lags behind the earthquake activity peak, meaning the number of liquid peaks is less than that of seismic waves, and the inconsistency is obvious. Furthermore, the increase in water level, and the time when the water level reaches the peak is advanced, with a value of 10%~14%.

(4) As the water level increases, the shaking amplitude of the liquid decreases, and the smaller the time from the peak to the trough (the period is negatively correlated with the water level). TLD uses the dynamic pressure generated during the sloshing of the liquid to provide shock absorption, and the greater the dynamic pressure, the more obvious the shock absorption effect. The results show that the hydrodynamic pressure of 0.7/L water level is the largest, and the shock absorption of the aqueduct structure is also better.

(5) Extracting from the wet mode of the aqueduct structure, including the main mode shape and self-resonating frequency, the natural frequency of the structure is the largest when the channel is empty. Whenever the water level increases, the modal coefficient of each order decreases and that of natural frequency decreases too.
